# Identification of m^6^A methylation-related genes in cerebral ischaemia‒reperfusion of Breviscapus therapy based on bioinformatics methods

**DOI:** 10.1186/s12920-023-01651-3

**Published:** 2023-09-05

**Authors:** Cheng Wan, Jingchun Pei, Dan Wang, Jihong Hu, Zhiwei Tang, Wei Zhao

**Affiliations:** 1 Department of Interventional Radiology, The First Affiliated Hospital of Kunming Medical University, Kunming, Yunnan, 650032 China; 2 Department of Neurosurgery, The First Affiliated Hospital of Kunming Medical University, Kunming, Yunnan, 650032 China; 3 Department of Organ Transplantation Centre, The First Affiliated Hospital of Kunming Medical University, Kunming, Yunnan, 650032 China

**Keywords:** Cerebral ischaemia-reperfusion, Breviscapine, m^6^A, Bioinformatics methods

## Abstract

**Background:**

Cerebral ischaemia‒reperfusion (I/R) frequently causes late-onset neuronal damage. Breviscapine promotes autophagy in microvascular endothelial cells in I/R and can inhibit oxidative damage and apoptosis. However, the mediation mechanism of breviscapine on neuronal cell death is unclear.

**Methods:**

First, transcriptome sequencing was performed on three groups of mice: the neuronal normal group (Control group), the oxygen-glucose deprivation/ reoxygenation group (OGD/R group) and the breviscapine administration group (Therapy group). Differentially expressed genes (DEGs) between the OGD/R and control groups and between the Therapy and OGD/R groups were obtained by the limma package. N^6^-methyladenosine (m^6^A) methylation-related DEGs were selected by Pearson correlation analysis. Then, prediction and confirmation of drug targets were performed by Swiss Target Prediction and UniProt Knowledgebase (UniProtKB) database, and key genes were obtained by Pearson correlation analysis between m^6^A-related DEGs and drug target genes. Next, gene set enrichment analysis (GSEA) and Ingenuity pathway analysis (IPA) were used to obtain the pathways of key genes. Finally, a circRNA-miRNA‒mRNA network was constructed based on the mRNAs, circRNAs and miRNAs.

**Results:**

A total of 2250 DEGs between the OGD/R and control groups and 757 DEGs between the Therapy and OGD/R groups were selected by differential analysis. A total of 7 m^6^A-related DEGs, including Arl4d, Gm10653, Gm1113, Kcns3, Olfml2a, Stk26 and Tfcp2l1, were obtained by Pearson correlation analysis. Four key genes (Tfcp2l1, Kcns3, Olfml2a and Arl4d) were acquired, and GSEA showed that these key genes significantly participated in DNA repair, e2f targets and the g2m checkpoint. IPA revealed that Tfcp2l1 played a significant role in human embryonic stem cell pluripotency. The circRNA-miRNA‒mRNA network showed that mmu_circ_0001258 regulated Tfcp2l1 by mmu-miR-301b-3p.

**Conclusions:**

In conclusion, four key genes, Tfcp2l1, Kcns3, Olfml2a and Arl4d, significantly associated with the treatment of OGD/R by breviscapine were identified, which provides a theoretical basis for clinical trials.

**Supplementary Information:**

The online version contains supplementary material available at 10.1186/s12920-023-01651-3.

## Background

Stroke is the leading cause of death in humans [1, 2]. Moreover, ischaemic stroke accounts for 60-80% of all brain strokes, with high morbidity, mortality and long-term severe disability [3]. Cerebral ischaemia‒reperfusion (I/R) injury is the major pathophysiological process that causes delayed neuronal injury [4]. The clinical treatment strategies are far from expected, with the secondary injury mechanism being complex [5] and still a huge health problem worldwide [6]. An increasing number of studies have shown that I/R injury activates a variety of cell death programs, such as acute necrosis, apoptosis, and autophagy [7], which lead to cognitive and memory dysfunction [8]. Novel and effective therapeutic targets require further exploration. However, N^6^-methyladenosine (m^6^A) is regarded as the most common and key regulator of mRNA modification [9] and affects the wide expression of genes in different pathophysiological processes by “writer” methyltransferases, “eraser” demethylases and “reader” proteins in eukaryotes [10]. Recent studies have demonstrated that m^6^A is involved in the occurrence and development of stroke, epilepsy and schizophrenia [11], but the relevant regulatory mechanism has not been fully elucidated.

Breviscapine is a traditional Chinese herbal medicine containing ≥ 90% scutellarin and ≤ 10% apigenin-7-O-glucronide that has been widely used to treat cerebrovascular disease in the past three decades [12] due to its functions of dilating cerebral vessels, reducing cerebral vascular resistance, increasing cerebral blood flow, improving microcirculation, decreasing blood viscosity and inhibiting platelet conglomeration [13–15]. In addition, breviscapine administration can promote autophagy of microvascular endothelial cells, exert antioxidative damage effects and attenuate neuronal cell apoptosis after I/R injury, which improves neurobehavioural functions [16]. However, the specific mechanism of breviscapine in I/R injury still needs to be further explored.

In this article, we aim to investigate the key genes modulated by breviscapine against cerebral I/R injury from the perspective of m^6^A methylation. According to the self-sequencing data from mice, four key m^6^A methylation-related genes (Tfcp2l1, Kcns3, Olfml2a and Arl4d) were identified, and these key genes have been considered to be significantly associated with the treatment of I/R injury by breviscapine, which provides a theoretical basis for clinical trials and a novel breakthrough point in the treatment of I/R.

## Methods

### Animals and cell culture

The experimental animals were SPF grade C57BL/6 mouse aged 1–3 days old, both male and female, provided by the Animal Experimental Center of Kunming Medical University (SYXK(Dian)K2020-0006), the number of qulitative qualification is SCK(Dian)K2020-0004. Neonatal mouse were placed in an anaesthetic induction box containing 3–4% isoflurane, which was inhaled for 3 min. All mouse were eventually sacrificed, and after separating the meninges, bilateral cerebral cortex was obtained. All experimental procedures were approved by the Animal Experimentation Ethics Review Committee of Kunming Medical University(approve number kmmu20221507). Primary cortical neuron culture was established by digesting cerebral cortex with 0.25% trypsin [17] and was divided into a control group, an OGD/R group, and a breviscapine administration group. The neurons were incubated with glucose-free RPMI 1640 medium and placed in an anaerobic chamber under an atmosphere of 95% N_2_ and 5% CO_2_ at 37 °C for 3 h. Then, the normal medium was replaced, and the cells were returned to normoxic conditions for reperfusion at 37 °C for 24 h, indicating that the OGD/R model was successfully established [18]. The breviscapine administration group was pretreated with 50µM breviscapine [19] before OGD and during reperfusion. Breviscapine were provided by the Kunming Longjin Pharmaceutical Co., Ltd ,Yunnan, China. With the content of 50 mg and the batch number is Z53020666 .

### RNA extraction and library construction sequencing

Total RNA was isolated and purified by TRIzol reagent following the instruction manual. The RNA amount and purity were quantified by a NanoDrop ND-1000. Agarose gel electrophoresis was used to verify the integrity of RNA (concentrations > 50 ng/µL, RIN values > 7.0, OD260/280 > 1.8, and total RNA > 1 µg satisfy downstream experiments). Oligo (dT) magnetic beads were used for specific capture of mRNA with polyA (polyadenylation) in them, and the captured mRNA was fragmented under high temperature conditions. Then, the fragmented RNA was reverse transcribed into cDNA. E. coli DNA polymerase I with RNase H was used for two-strand synthesis. These compound doublets of DNA and RNA were converted into DNA doublets, dUTP solution was incorporated into the doublets at the same time, and the ends of the double-stranded DNA were complemented to flat ends. An additional base was added to each end to enable ligation with a linker with a T base at the end, and its fragment size was screened and purified using magnetic beads. The second strand was digested with UDG enzyme, and then PCR was performed to form a library with a fragment size of 300 bp ± 50 bp. Finally, we used Illumina NovaSeq™ 6000 to perform double-end sequencing according to standard practice.

### Data sources

In this study, transcriptome sequencing was performed on three groups of mice: the neuronal normal group (Control group), the cerebral ischaemia and reperfusion group (OGD/R group) and the breviscapine administration group (Therapy group), with 7 mice in each group. Seven pairs of mice were sequenced for mRNA transcriptome analysis, and 3 pairs were sequenced for whole transcriptome analysis. Twenty-one m^6^A regulatory factors were obtained from published literature [20].

### The acquisition of differentially expressed genes (DEGs)

The mRNA transcriptome data and whole transcriptome sequencing data were used for differential analysis. The limma package (version 3.48.3) [21] was used to obtain the differentially expressed genes (DEGs) between the OGD/R and control groups and between the Therapy and OGD/R groups. Then, the upregulated DEGs between the OGD/R group and control group and the downregulated DEGs between the Therapy group and OGD/R group were intersected to obtain DEGs. The ggplot2 package (version 3.3.5) [22] and the pheatmap package (version 1.0.12) were used to plot the volcano plots and heatmaps, respectively.

### Construction of a protein‒protein interaction (PPI) network

According to the expression of 21 m^6^A regulatory factors, the Wilcoxon test was used to compare the m^6^A between the OGD/R and control groups and between the Therapy and OGD/R groups. The box plot was drawn using the ggpubr package for visualization. Then, in the OGD/R and control groups and the Therapy and OGD/R groups, the correlation between differentially expressed m^6^A regulatory factors and the DEGs was calculated by Pearson correlation coefficient (|cor| > 0.5 and p.value < 0.01). Then, the m^6^A-related DEGs between the OGD/R and control groups crossed with the m^6^A-related DEGs between the Therapy and OGD/R groups. Finally, the PPI network of m^6^A regulatory factors and m^6^A-related DEGs was constructed.

### Functional enrichment analysis and drug target prediction

Gene Ontology (GO) and Kyoto Encyclopedia of Genes and Genomes (KEGG) functional enrichment analyses of m^6^A-related DEGs were performed by clusterprofiler (version 4.0.2) [23] (p.adjust < 0.05 and count ≥ 1). The Swiss Target Prediction database (http://www.swisstargetprediction.ch/) and UniProt Knowledgebase (UniProtKB) database (http://www.uniprot.org) were used to predict the target compounds and target genes of breviscapine. Then, the target compound and target gene regulatory network was constructed by Cytoscape.

### Identification and gene set enrichment analysis (GSEA) of key genes

The correlation between the m^6^A-related DEGs and drug target genes was performed by Pearson correlation coefficient, and the significant drug target genes were selected as key genes (|cor| > 0.7, p value < 0.01). Then, the PPI network was constructed by the drug target genes and key genes. The ClusterProfiler (version 4.0.2) package [23] and org.Hs.eg.db package (version 3.13.0) were used to explore the related pathways and molecular mechanisms of key genes.

### Ingenuity pathway analysis (IPA) and construction of a regulatory network

IPA (www.ingenuity.com) was performed to acquire the signalling pathways of key genes. Then, we used IPA to analyse the upstream regulatory factors and downstream target genes of key genes, and we found the regulatory relationship of key genes. Transcription factors (TFs) in mice were downloaded from the AnimalTFDB database (http://bioinfo.life.hust.edu.cn/AnimalTFDB#!/), and the expression levels of TFs were obtained from the self-sequencing dataset. The Psych package was used to calculate the Pearson correlation coefficient between TFs and key genes. The TFs were selected to construct the TF-mRNA network (|cor| ≥ 0.8 and P ≤ 0.01).

### Construction of a ceRNA network

First, differentially expressed lncRNAs (DELs) between the OGD/R and control groups and between the Therapy and OGD/R groups were obtained by the limma package (version 3.48.3) (|logFC| ≥ 0.5, p value < 0.05) in 7 pairs of mRNA transcriptomes and 3 pairs of whole transcriptome samples. Then, the downregulated DELs between the OGD/R and control groups and upregulated DELs between the Therapy and OGD/R groups were intersected to obtain DELs. The limma package (version 3.48.3) was used to obtain the differentially expressed circRNAs (DECs) between the OGD/R and control groups and between the Therapy and OGD/R groups in the 3 pairs of whole transcriptome samples. Then, DECs were obtained with the intersection of downregulated DECs in the OGD/R and control groups and upregulated DECs in the Therapy and I/R groups. Heatmaps and volcano plots were plotted by the pheatmap (version 1.0.12) and ggplot2 (version 3.3.5) [22] packages, respectively. Next, the target binding miRNAs of key genes and the miRNAs of intersecting DECs were predicted based on the ENCORI database (https://starbase.sysu.edu.cn/). The circRNA-miRNA‒mRNA regulatory network was constructed by the common miRNA of circRNA and mRNA, and Cytoscape was used to visualize the network. In addition, the target genes of codifferentially expressed lncRNAs were predicted by the coexpression relationship, and the correlation between codifferentially expressed lncRNAs and key genes was calculated using Pearson correlation analysis. Finally, coexpressed lncRNAs and mRNAs were selected to construct the lncRNA‒mRNA regulatory network (|cor| ≥ 0.7 and p value ≤ 0.01), and the key gene regulation-pathway network was created based on the predicted ceRNA networks and lncRNAs of key genes.

### Results

#### Analysis of differential expression

**Fig. 1:**
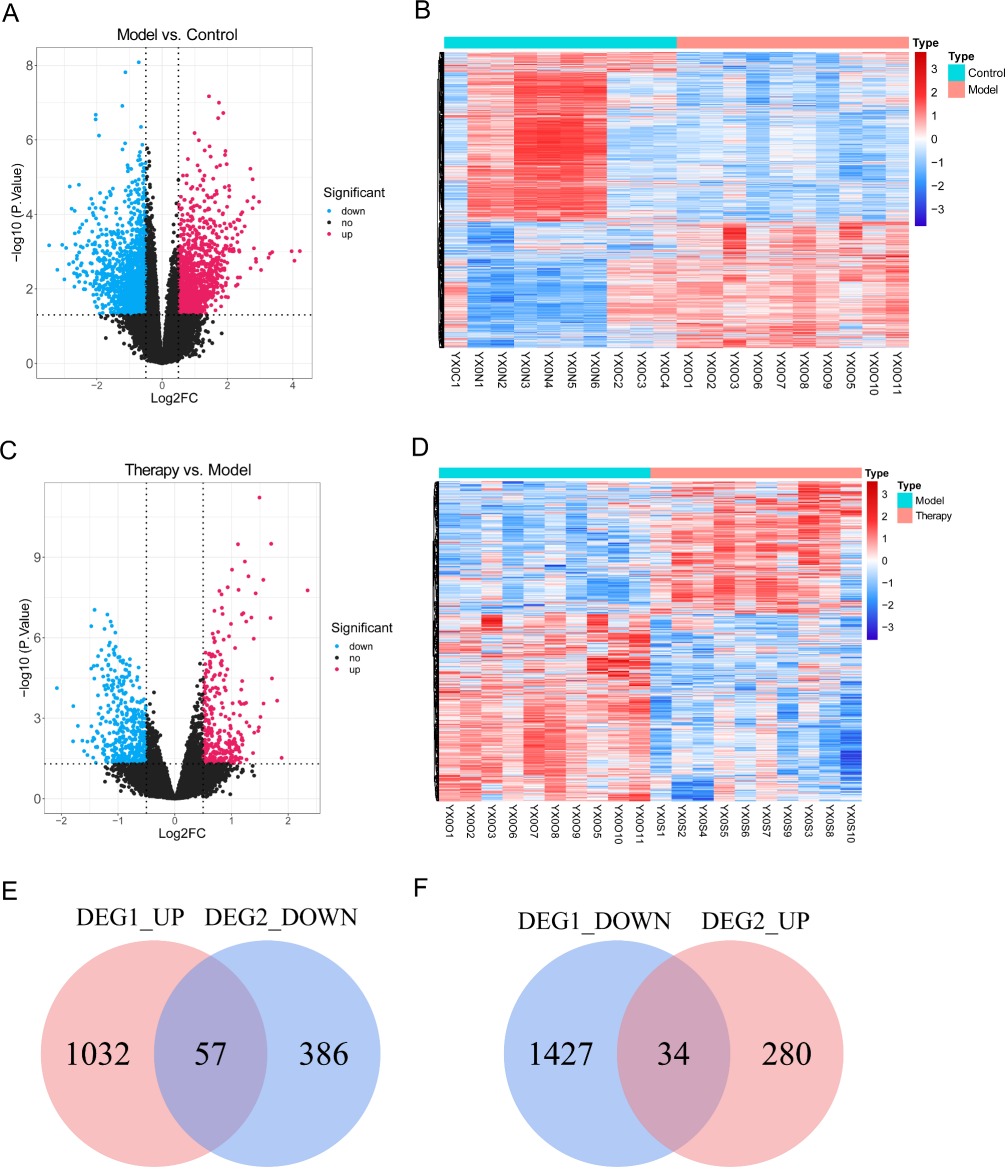
Differential expression analysis. **(A**, **B)** Differentially expressed genes (DEGs) between the OGD/R and control groups in a volcano plot **(A)** and heatmap **(B)**. **(C**, **D)** DEGs between the Therapy and OGD/R groups in the volcano plot **(C)** and heatmap **(D)**. **(E**-**F)** Venn diagram identifying overlapping DEGs between different groups

A total of 2250 DEGs between the OGD/R and control groups were selected, including 1089 upregulated and 1461 downregulated DEGs (Fig. 1A, B). There were 757 DEGs between the Therapy and OGD/R groups (314 upregulated and 443 downregulated) (Fig. 1C, D). Fifty-seven upregulated DEGs and 34 downregulated DEGs were obtained between the OGD/R and control groups (Fig. 1E, F).

### Screening of m6A regulatory factor-related genes

**Fig. 2:**
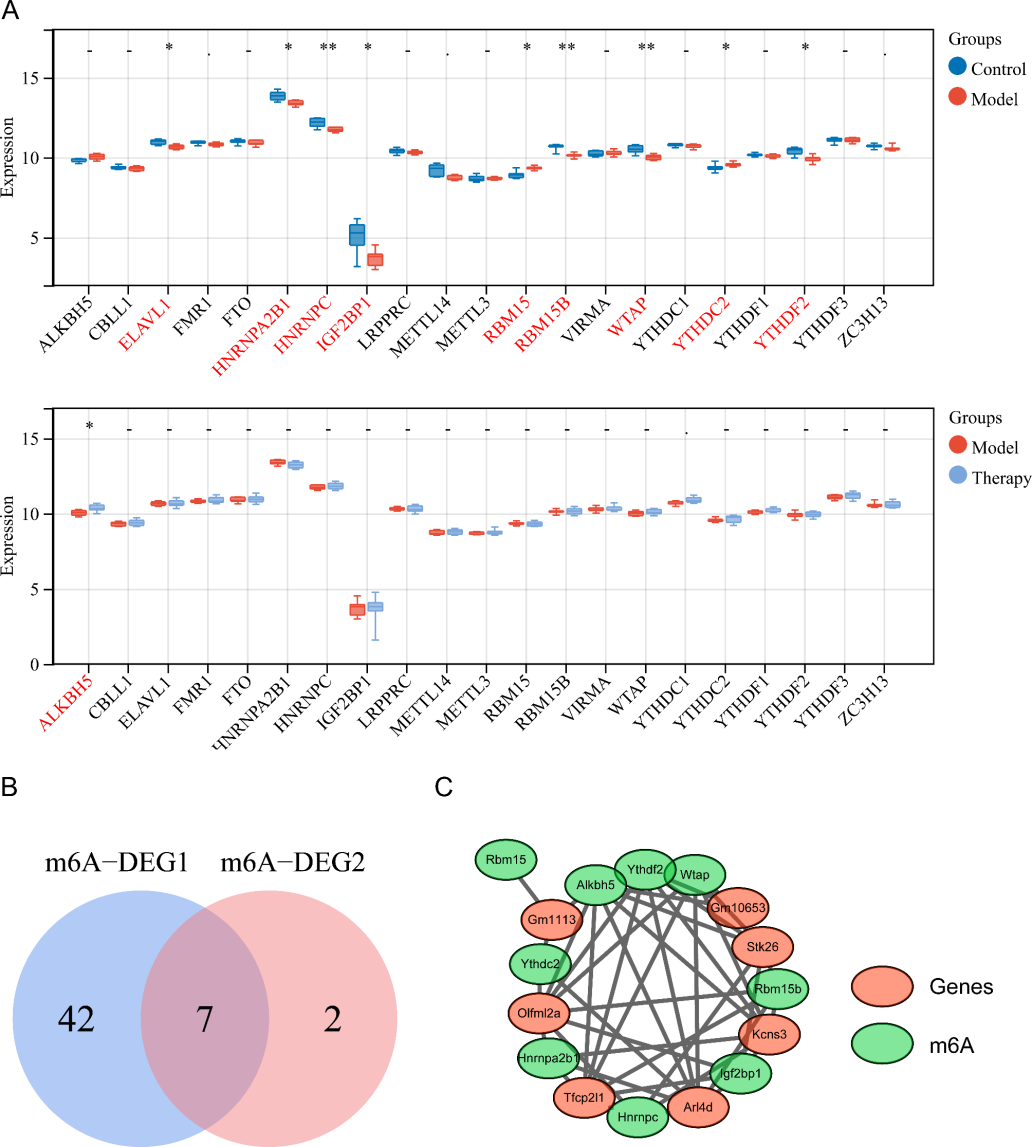
Identification of m^6^A regulatory factor-related genes. **(A)** Expression patterns of m^6^A regulatory factors in the OGD/R and control groups (top) and the Therapy and OGD/R groups (bottom). **(B)** Venn diagram identifying overlapping m6A-related DEGs. **(C)** Protein‒protein interaction (PPI) diagram of m^6^A- and m^6^A-related differentially expressed genes

Nine m^6^A regulatory factors had significant differences (ELAVL1, HNRNPA2B1, HNRNPC, IGF2BP1, RBM15, RBM15B, WTAP, YTHDC2, and YTHDF2) between the OGD/R and control groups. One m^6^A regulatory factor (ALKBH5) was remarkably different between the Therapy and OGD/R groups (Fig. 2A). Forty-nine m^6^A-related DEGs between the OGD/R and control groups and 9 m^6^A-related DEGs between the Therapy and OGD/R groups were obtained by Pearson analysis. Seven m^6^A-related DEGs were obtained after intersection, including Arl4d, Gm10653, Gm1113, Kcns3, Olfml2a, Stk26, and Tfcp2l1 (Fig. 2B). There were 7 m^6^A-related DEGs connected with 8 m^6^A regulatory factors (Fig. 2C).

### Functional enrichment of m^6^A-related DEGs and breviscapine drug target prediction

**Fig. 3:**
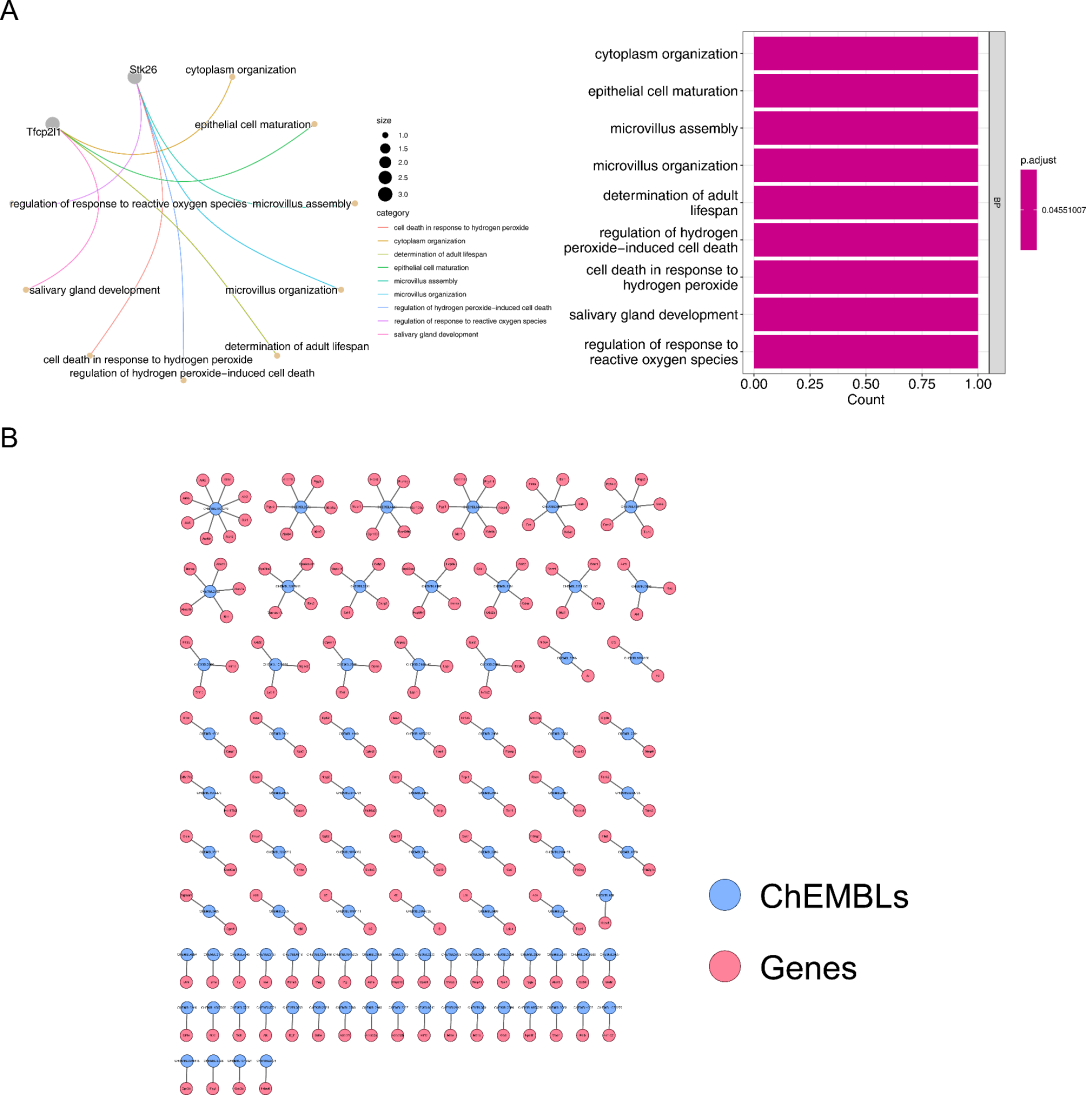
Functional enrichment of m^6^A-related DEGs and breviscapine drug target prediction. **(A)** GO enrichment analysis of differentially expressed genes and enrichment of biological processes. **(B)** Target compounds of the breviscapine-target gene regulatory network

There were 9 biological processes (BPs) of GO enriched for 7 m^6^A-related DEGs, such as cytoplasm organization, epithelial cell maturation and microvillus assembly (Fig. 3A). Eighty-six target compounds and 176 target genes of breviscapine were obtained by the Swiss Target Prediction and UniProtKB databases, and CHEMBL1075275 of the target compounds regulated 8 genes, including Aurkb, Aik2, Aim1, Airk2, Ark2, Stk1, Stk12 and Stk5 (Fig. 3B).

### Acquisition of key genes and gene set enrichment analysis (GSEA)

**Fig. 4:**
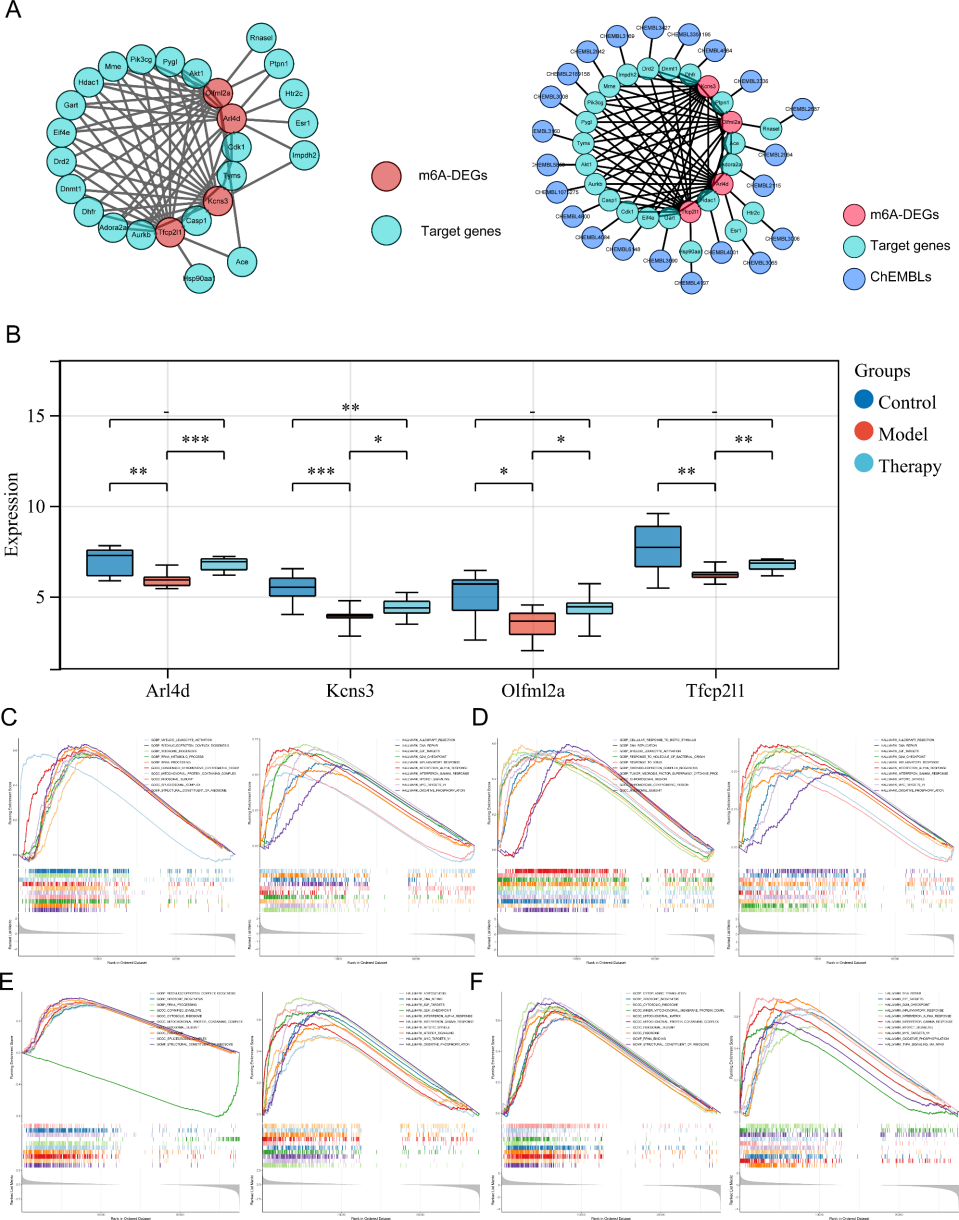
Acquisition of key genes and gene set enrichment analysis (GSEA). **(A)** PPI network for key genes and drug target genes (A1). ChEMBLs regulated the key gene‒target gene-compound network (A2). **(B)** Four key gene expression patterns. (C-F) GSEA-Hallmark and GSEA-GO enrichment analysis of 4 key genes

Four key genes, Tfcp2l1, Kcns3, Olfml2a and Arl4d, were obtained by correlation analysis (Supplementary Table 1). It is worth noting that 15 drug target genes (Pik3cg, Pygl, Akt1, Cdk1, Tyms, Casp1, Aurkb, Adora2a, Dhfr, Dnmt1, Drd2, Eif4e, Gart, Hdac1 and Mme) were connected with 4 key genes in the PPI network, and CHEMBL2687 regulated Olfml2a by Rnasel in the key gene‒target gene-compound network (Fig. 4A). The expression of the 4 key genes in the OGD/R groups was the lowest among the three groups (Fig. 4B). In the GO functional enrichment, Tfcp2l1 and Olfml2a were mainly enriched in ribosome biogenesis, rRNA processing and ribosomal subunit; Kcns3 participated in cellular response to biotic stimulus, DNA replication and ribosomal subunit; and Arl4d was involved in cytoplasmic translation, ribosome biogenesis and ribosomal subunit. Hallmark enrichment showed that the 4 key genes including Tfcp2l1, Kcns3, Olfml2a and Arl4d, mainly participated in DNA repair, e2f targets and the g2m checkpoint (Fig. 4C F**).**

### IPA enrichment and construction of the TF-mRNA regulatory network

**Fig. 5:**
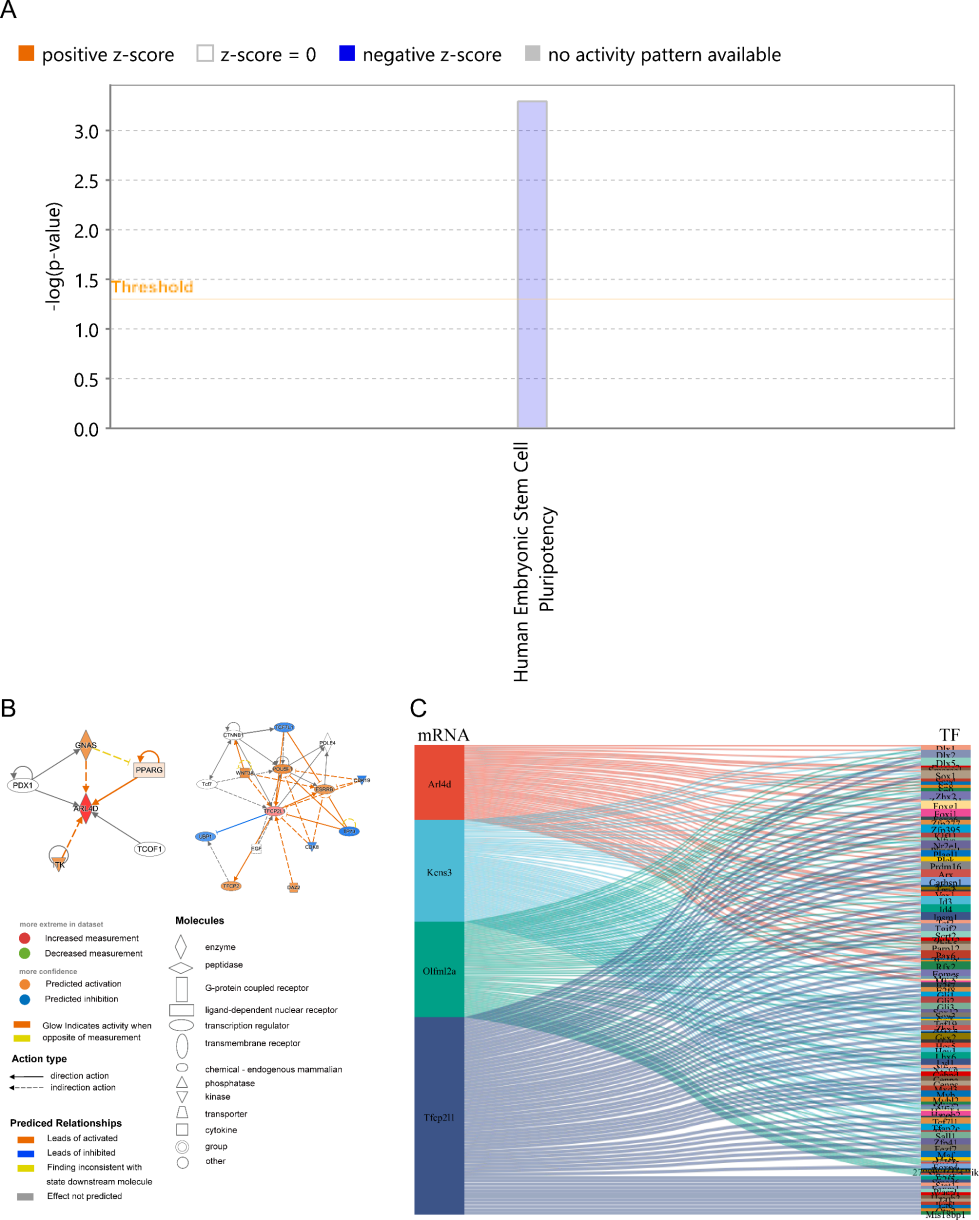
Ingenuity pathway analysis (IPA) enrichment and construction of the TF-mRNA regulatory network. **(A)** IPA analysis pathway results. **(B)**. Key gene regulatory networks for Arl4d and Tfcp2l1. **(C)** The TF-mRNA regulatory network of key genes

Tfcp2l1 was significantly enriched in human embryonic stem cell pluripotency (Fig. 5A). In Tfcp2l1 regulatory networks, Tfcp2l1 was regulated by POU5F1, and ESRRB and TFCP2 were directed to regulate Tfcp2l1. Arl4d was regulated by PPARG in the Arl4d regulatory network (Fig. 5B). A total of 1482 TFs were obtained from the self-sequencing dataset. A total of 109 TFs were selected by correlation analysis, and a TF-mRNA regulatory network was constructed by the TFs (Fig. 5C, Supplementary Table 2).

### Construction of circRNA-miRNA‒mRNA and lncRNA‒mRNA networks

**Fig. 6:**
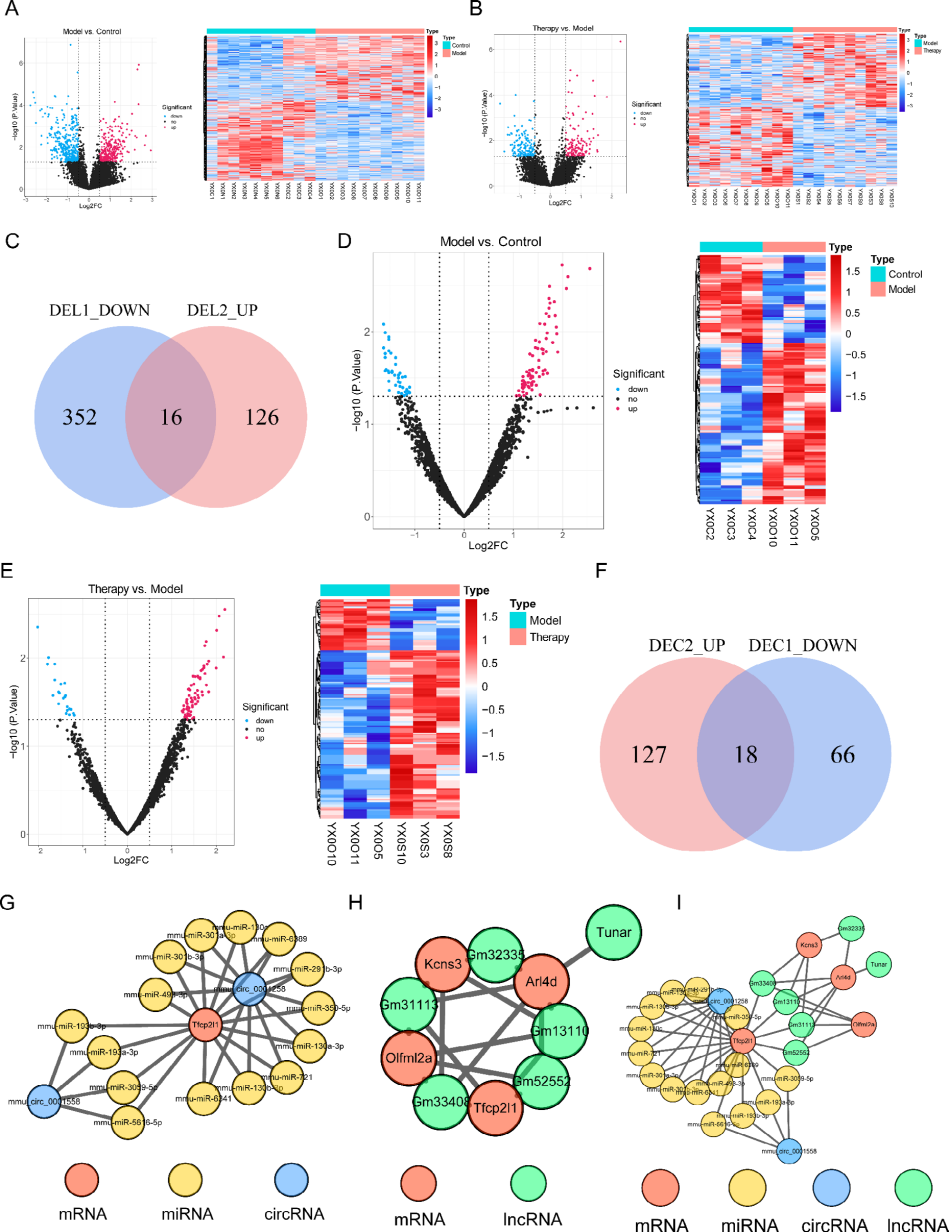
Construction of circRNA-miRNA‒mRNA and lncRNA‒mRNA networks. **(A**, **B)** Differential expression analysis of lncRNAs between the model and control samples **(A)** and the Therapy and OGD/R groups **(B)**. **(C)** Venn diagram of differential lncRNAs. **(D**, **E)** Differential expression of circRNAs between the model and control groups **(D)** and the Therapy and OGD/R groups **(E)**. **(F)** Venn diagram of differential circRNAs. **(G)** The circRNA-miRNA‒mRNA network of key genes. **(H)** The lncRNA‒mRNA network of key genes. **(I)** The regulatory-pathway network of key genes

There were 667 DELs between the OGD/R and control groups, including 299 upregulated and 368 downregulated lncRNAs (Fig. 6A). A total of 295 DELs were obtained between the Therapy and OGD/R groups (142 upregulated and 153 downregulated) (Fig. 6B). Sixteen DELs were obtained by the intersection of downregulated DELs between the OGD/R and control groups and upregulated DELs between the Therapy and I/R groups (Fig. 6C, Supplementary Table 3). There were 238 DECs obtained between the OGD/R and control groups, of which 154 DECs were upregulated and 84 DECs were downregulated (Fig. 6D). In the Therapy and OGD/R groups, 189 DECs were obtained, including 145 upregulated and 44 downregulated DECs (Fig. 6E). Eighteen DECs were obtained by taking the intersection of downregulated DECs between the OGD/R and control groups and upregulated DECs between the Therapy and OGD/R groups (Fig. 6F, Supplementary Table 4). The regulatory network of circRNA-miRNA‒mRNA was constructed based on 2 circRNAs, 15 miRNAs and 1 mRNA. The mmu_circ_0001258 circRNA regulated Tfcp2l1 by 11 miRNAs, including mmu-miR-493-3p, mmu-miR-301b-3p, mmu-miR-301a-3p, mmu-miR-130c, mmu-miR-6389, mmu-miR-291b-3p, mmu-miR-350-5p, mmu-miR-130a-3p, mmu-miR-721, mmu-miR-130b-3p and mmu-miR-6341 (Fig. 6G). Four key genes, Tfcp2l1, Kcns3, Olfml2a and Arl4d, were regulated by 5 lncRNAs (Gm33408, Gm52552, Gm13110, Gm32335, and Gm31113) (Fig. 6H). Only Tfcp2l1 was co-regulated by miRNA and lncRNA (Fig. 6I).

## Discussion

Although rapid restoration of blood supply is the best treatment for ischaemic stroke, secondary brain tissue damage inevitably emerges in the reperfusion territory [24, 25]. Growing evidence shows that hypoxia, oxidative stress, and the inflammatory response are common neurological events in ischaemic stroke [26, 27]. The dysregulation of m6A modification is closely associated with cancer and cerebrovascular diseases [28, 29]. However, posttranscriptional m^6^A modification can influence the expression of key proteins and could provide new insight into treating cerebral I/R injury [30, 31]. Breviscapine administration for ischaemic stroke is effective and reliable [12] and improves neurological function and reduces the cerebral infarction area based on neuroprotective and anti-coagulation effects [32]. Moreover, accumulating evidence has proven that breviscapine can suppress proinflammatory cytokine (IL-6, IL-1β, and TNF-α) expression, decrease reactive oxygen species (ROS) levels, and attenuate massive nerve cell death and brain tissue destruction after cerebral I/R [33]. However, the role of m^6^A with breviscapine in cerebral I/R injury has not been studied. In this article, we identified four m^6^A methylation-related genes (Tfcp2l1, Kcns3, Olfml2a and Arl4d) significantly associated with the treatment of OGD/R by breviscapine, which plays a foundation for further exploring the mechanism of breviscapine neuronal protective effects for cerebral I/R.

However, studies have shown that Tfcp2l1 is a downstream target of the canonical Wnt/β-catenin, STAT3 and FGF/MEK signalling pathways and plays a pivotal role in promoting embryonic stem cell self-renewal [34, 35]. In addition, the lower Kcns3 levels provide a new molecular mechanism of neuron dysfunction in schizophrenia, which encodes potassium channel-associated subunits [36]. Olfml2a is a key regulatory protein acting downstream of AP-1 [37]. Knockdown of Olfml2a in glioma cells could promote apoptosis and inhibit the Wnt/β-catenin signalling pathway [38]. Studies have shown that Arl4d is involved in vesicle trafficking, organelle structure, cytoskeleton organization, modulation of cell migration [39] and microtubule growth [40]. In conclusion, the molecular functions and regulatory mechanisms of four key genes (Tfcp2l1, Kcns3, Olfml2a, Arl4d) have not been reported in cerebral I/R injury, and further research is needed.

Research has also shown that DNA hypomethylation can promote learning and memory recovery in cerebral I/R rat models [41]. Our results demonstrated that Tfcp2l1, Kcns3, Olfml2a and Arl4d were significantly downregulated in cerebral OGD/R models and the control group. Additionally, upregulation with breviscapine administration most likely plays a protective role against cerebral I/R and thus provides novel treatment insights into cerebral I/R injury. However, the mechanism is poorly understood, and no reports have focused on this topic.

A study showed that ischaemia preconditioning, which resulted in an overall decrease in global DNA methylation in neuronal cultures, may improve tolerance to cell death [42]. The levels of m^6^A modification were significantly increased after neuronal OGD/R and rat MCAO treatment, which regulated neuronal synaptic plasticity, axonal growth, apoptosis, learning and memory, and stress responses induced by I/R [43]. In recent years, m^6^A-modified lncRNAs have received extensive attention. m^6^A modification of RNA plays an important role in pathophysiological processes in the central nervous system, in which lncRNAs are key biomarkers in cerebral ischaemic disease [44], and the m^6^A modification status in lncRNAs may control the biological functions of lncRNAs [45]. Noncoding RNAs are considered novel therapeutic targets for ischaemic stroke [46, 47]. However, five lncRNAs (Gm33408, Gm52552, Gm13110, Gm32335 and Gm31113) and two circRNAs (mmu_circ_0001258 and mmu_circ_0001558) were identified, and their specific functions and roles have not been reported, which may be a new entry point for cerebral I/R injury.

Owing to the limitations of current experiments, elucidating the dynamic regulatory mechanisms of individual m^6^A sites and their molecular functions remains challenging [48]. We analysed the sequencing results of primary neurons by bioinformatics methods and showed that four key genes were significantly decreased in the model group but significantly increased in the treatment group. We speculate that breviscapine can regulate the levels of m^6^A methylation during cerebral I/R, which may be a potential new mechanism for treating cerebral I/R injury. These genes were also analysed by ceRNA network, lncRNA‒mRNA coexpression network and regulation-pathway, which showed that key genes play a critical role in the regulation of m^6^A with cerebral ischaemia reperfusion, especially in DNA repair, e2f targets and g2m checkpoint. Of course, several limitations in our study should be noted, such as the sample size being relatively small. Further molecular experimental research and clinical applications are needed, and we will continue to pay more attention to the role of these genes.

There is no evidence of direct interaction among the four key genes, but the regulation-pathway network of the key genes suggests that the four key genes may interact with each other indirectly through the action of IncRNAs (e.g., Gm33408, Gm13110, Gm31113, and Gm52552), but the specific regulatory mechanism still needs to be further investigated. The database was also not searched for data related to the expression of the four genes after ischaemic stroke. Additionally, the expression of the four key genes was decreased after neuronal OGD/R and upregulated after the use of breviscapine, which may be involved in neuronal damage and repair after cerebral I/R injury and is also a sensitive biomarker for evaluating the efficacy of breviscapine. The results of the present study may be helpful for clinical trials and provide new perspectives for the treatment of ischaemic stroke.

## Conclusion

In summary, this study demonstrated that breviscapine could regulate the gene expression levels of m^6^A in neuronal I/R injury. This is the first study to identify these genes, which may play an important role in cerebral I/R injury. Additionally, prediction of the drug target of breviscapine might provide novel potential therapeutic targets for cerebral ischaemia stroke.

### Electronic supplementary material

Below is the link to the electronic supplementary material.

Supplementary Material 1

Supplementary Material 2

Supplementary Material 3

Supplementary Material 4

Supplementary Material 5

## Data Availability

The datasets generated and/or analysed during the current study are available in the [ArrayExpress] repository, [Persistent web link: https://www.ebi.ac.uk/biostudies/arrayexpress/studies/E-MTAB-13017?key=0d170feb-c91b-4582-b4c8-8200f01c4b43].

## List of abbreviations

I/R: Ischaemia‒reperfusion
DEGs: Differentially expressed genes
m6A: N6-methyladenosine
UniProtKB: UniProt Knowledgebase
GSEA: Gene set enrichment analysis
IPA: Ingenuity pathway analysis
OGD: Oxygen-glucose deprivation
GO: Gene Ontology
KEGG: Kyoto Encyclopedia of Genes and Genomes
TF: Transcription factors
DELs: Differentially expressed lncRNAs
DECs: Differentially expressed circRNAs
