# Supplementary material for: Identification of m6A methylation-related genes in cerebral ischaemia‒reperfusion of Breviscapus therapy based on bioinformatics methods
Source: BMC Med Genomics. 2023 Sep 5;16:210. doi: 10.1186/s12920-023-01651-3 (PMC10478429; doi:10.1186/s12920-023-01651-3)
Supplement: Supplementary file 5 — Supplementary Material 5 [file 12920_2023_1651_MOESM5_ESM.docx]

**Supplementary Table 1** Identification of key genes by correlation analysis.

**Supplementary Table 2** The TF-mRNA regulatory network.

**Supplementary Table 3** 16 differentially expressed lncRNAs (DELs) obtained by overlapping the down-regulation DELs between OGD/R and Control groups and up-regulation DELs between Therapy and I/R groups.

**Supplementary Table 4** 18 differentially expressed circRNAs (DECs) obtained by taking the intersection of down-regulation DECs between OGD/R and Control groups and up-regulation DECs between Therapy and OGD/R groups.
